# Sex-specific considerations in chronic osteoarthritis pain research: commentary on Pacheco-Barrios et al. (2025)

**DOI:** 10.1016/j.bjane.2025.844694

**Published:** 2025-10-27

**Authors:** Isra Panhwer, Anzalna Bashir, Safia Panhwer, Kalpana Singh

**Affiliations:** a Liaquat University of Medical & Health Sciences, Jamshoro, Sindh, Pakistan; b Hamad Medical Corporation, Doha, Qatar

**Keywords:** Sex-specific, Osteoarthritis, Pain

Dear Editor,

We read with great interest the article titled *“The role of biological sex in neurophysiological associations of patients with chronic osteoarthritis pain: a prospective cross-sectional study”* by Pacheco-Barrios et al. (2025),^1^ recently published in *The Brazilian Journal of Anesthesiology*. This study addressed a crucial gap by examining whether biological sex influences the associations between clinical, pain-related, and neurophysiological outcomes in patients with chronic knee Osteoarthritis (OA) pain. While the research makes a significant contribution to the field, several methodological and interpretive issues merit further discussion.

Despite aiming to explore the role of biological sex, the sample was notably unbalanced, with a predominance of female participants (*n* = 94) compared to males (*n* = 19) out of a total of 113. As noted by Bartley and Fillingim,^2^ uneven sex distribution in pain research may obscure actual sex-based differences, possibly leading to overgeneralizations or misinterpretations. Future research should aim for more representative sampling to enable robust sex-based comparisons.

The study was conducted at a single rehabilitation hospital in Brazil with a relatively small sample size. This constrains the generalizability of findings to broader populations with diverse demographic and clinical characteristics. As Woitowich et al.^3^ have emphasized, sex-disaggregated analyses require adequately powered and representative samples to yield valid conclusions about sex differences in biomedical research.

The authors suggest that the predominance of postmenopausal women may have mitigated hormonal variability. However, the absence of specific data on menopausal status, hormone replacement therapy, or gonadal hormone levels is a critical limitation. Without controlling for these variables, it becomes difficult to distinguish between biological sex effects and hormonal influences. This is particularly relevant when analyzing neurophysiological metrics such as EEG or TMS, which are sensitive to fluctuations in estrogen and testosterone. Prior work has shown that the menstrual cycle significantly impacts stress response circuitry and neural activation in women.^4^

The recent study by McCabe et al. (2025)^5^ highlights sex-specific diagnostic models for knee osteoarthritis and the importance of incorporating individualized variables, including hormonal history. Their findings support a broader framework that integrates not just demographics and clinical variables but also neurophysiological biomarkers such as quantitative EEG and functional MRI to better understand the central mechanisms underlying pain. Furthermore, expanding such models to diverse global cohorts and targeting early interventions may enhance diagnostic accuracy, clinical equity, and prevention strategies.

In conclusion, while Pacheco-Barrios et al. make a commendable effort to address sex-related differences in chronic osteoarthritis pain, we urge future studies to prioritize balanced sampling, consider hormonal status variables, and explore integrative models that reflect both peripheral and central contributors to pain. Such efforts will advance the path toward truly personalized pain management.

After a few attempts to contact the original authors of the study, we did not receive a response.

## Authors’ contributions

Isra Panhwer: Contributed to critique on lack of hormonal and menopausal data.

Anzalna Bashir: Contributed to critique on uneven sex distribution and limited sample size, and she is also our corresponding author.

Safia Panhwer: Contributed to emphasize the importance of incorporating sex-specific diagnostic models in chronic pain research to improve clinical relevance.

Kalpana Singh: Revised the entire article and modified it.

## Conflicts of interest

The authors declare no conflicts of interest.
